# Case Report: Cholestatic liver disease in the course of erythropoietic protoporphyria associated with renal hypodysplasia and atrial septal defect

**DOI:** 10.3389/fped.2025.1504181

**Published:** 2025-02-11

**Authors:** Patryk Lipiński, Agnieszka Lipniacka, Maja Klaudel-Dreszler, Lidia Ziółkowska, Grażyna Kostrzewa, Edyta Odnoczko, Robert Wasilewski, Rafał Płoski, Anna Tylki-Szymańska

**Affiliations:** ^1^ Institute of Clinical Sciences, Maria-Skłodowska-Curie Medical Academy, Warsaw, Poland; ^2^ Department of Haemostasis and Metabolic Diseases, Institute of Haematology and Transfusion Medicine, Warsaw, Poland; ^3^ Department of Gastroenterology, Hepatology, Feeding Disorders and Pediatrics, The Children’s Memorial Health Institute, Warsaw, Poland; ^4^ Department of Cardiology, The Children’s Memorial Health Institute, Warsaw, Poland; ^5^ Department of Medical Genetics, Medical University of Warsaw, Warsaw, Poland; ^6^ Department of Disorders of Hemostasis and Internal Medicine, Institute of Haematology and Transfusion Medicine, Warsaw, Poland; ^7^ Department of Pediatrics, Nutrition and Metabolic Diseases, The Children’s Memorial Health Institute, Warsaw, Poland

**Keywords:** erythropoietic protoporphyria, cholestasis, atrial septal defect, renal hypodysplasia, children

## Abstract

Erythropoietic protoporphyria (EPP) is an autosomal recessive disorder of the heme biosynthesis pathway caused by pathogenic variants in *FECH* gene resulting in a decreased activity of ferrochelatase. Liver involvement is observed in 5%–20% of patients harbouring loss-of-function *FECH* variants and its manifestations are heterogeneous, ranging from mildly elevated liver transaminases, cholelithiasis to severe acute cholestatic hepatitis/liver failure. This paper presents the case of a Polish infant with EPP associated with two novel missense *FECH* variants accompanied by other congenital anomalies, namely atrial septal defect and renal hypodysplasia. Progressive cholestatic liver disease (with subsequent congestive heart failure) was observed in the course of EPP. Erytropoietic protoporphyria should be considered in patients with hepatosplenomegaly and cholestasis accompanied by skin damage. The natural history of liver disease in the course of EPP could be determined by other factors, like the co-existence of congenital anomalies.

## 1 Background

Erythropoietic protoporphyria (EPP, #177000) along with X-linked protoporphyria (XLP, #300752) represent two clinically indistinguishable types of protoporphyria (PP). EPP is an autosomal recessive disorder of the heme biosynthesis pathway caused by pathogenic variants in *FECH* gene resulting in a decreased activity of ferrochelatase (1–4). XLP is a less common (2%–10% patients of PP) condition with a similar clinical phenotype resulting from gain-of-function mutations in the aminolevulinic acid synthase 2 gene (*ALAS2*) (1–4). Both these forms of PP are characterized with protoporphyrin IX (PPIX) accumulation in erythroid cells and other tissues (1–4). Clinically, they present with acute painful phototoxicity on sun-exposed skin areas starting in infancy or childhood. Liver involvement is observed in a minority of patients and its manifestations are diverse, ranging from mildly elevated liver transaminases, cholelithiasis to severe acute cholestatic hepatitis/liver failure (5–7). EPP and XLP are diagnosed based on the biochemical analysis of total erythrocyte protoporphyrin (ePP), while the metal-free protoporphyrin IX (PPIX) is predominantly found in EPP (4, 8, 9). The molecular analysis of corresponding genes (FECH/ALAS2) confirms the clinical and biochemical diagnosis. However, the diagnosis of PP could be delayed because of the insufficient awareness among doctors, the clinical variability of its manifestation and the rarity of disease.

The aim of this manuscript was to present the first known diagnosed case in Poland of pediatric patient with EPP associated with two novel missense *FECH* variants, emphasize the co-existence of EPP with other congenital anomalies and report the clinical outcome of progressive cholestatic liver disease in the course of EPP.

## 2 Methods

### 2.1 Genetic analysis

DNA from proband and her parents was retrieved from peripheral blood and extracted with a standard protocol. Library preparation for the Whole Exome Sequencing (WES) was performed on proband's DNA sample with Twist Human Core Exome spiked-in with Twist mtDNA Panel, Twist RefSeq Panel and Custom Panel covering variants located in noncoding regions that have been linked to clinical phenotypes according to the ClinVar database (Twist Bioscience, San Francisco, CA, USA). Enriched library was paired-end sequenced (2 × 100 bp) on NovaSeq 6000 (Illumina, San Diego, CA, USA) to obtain 77 960 870 reads resulting in mean depth of 76.52× (98.6% of target bases were covered at a minimum of 20×, whereas 99.1% had coverage of min. 10×). Bioinformatic analysis of raw WES data and variants prioritization were performed as previously described (10). Reads were aligned to the hg38 reference genome sequence and visualized by Integrative Genomic Viewer. Presence of the selected variant was confirmed and established by amplicon deep sequencing (ADS) in the proband and her parents.

### 2.2 Biochemical analysis

Blood for free and zinc protoporphyrin measurement was collected into a blood collection tube with EDTA anticoagulant and stool sample for the measurement of porphyrins and their isomers was collected into an appropriate container. Light-protected collected samples were extracted and analysed as previously described (11).

## 3 Patient's presentation

**Figure 1:**
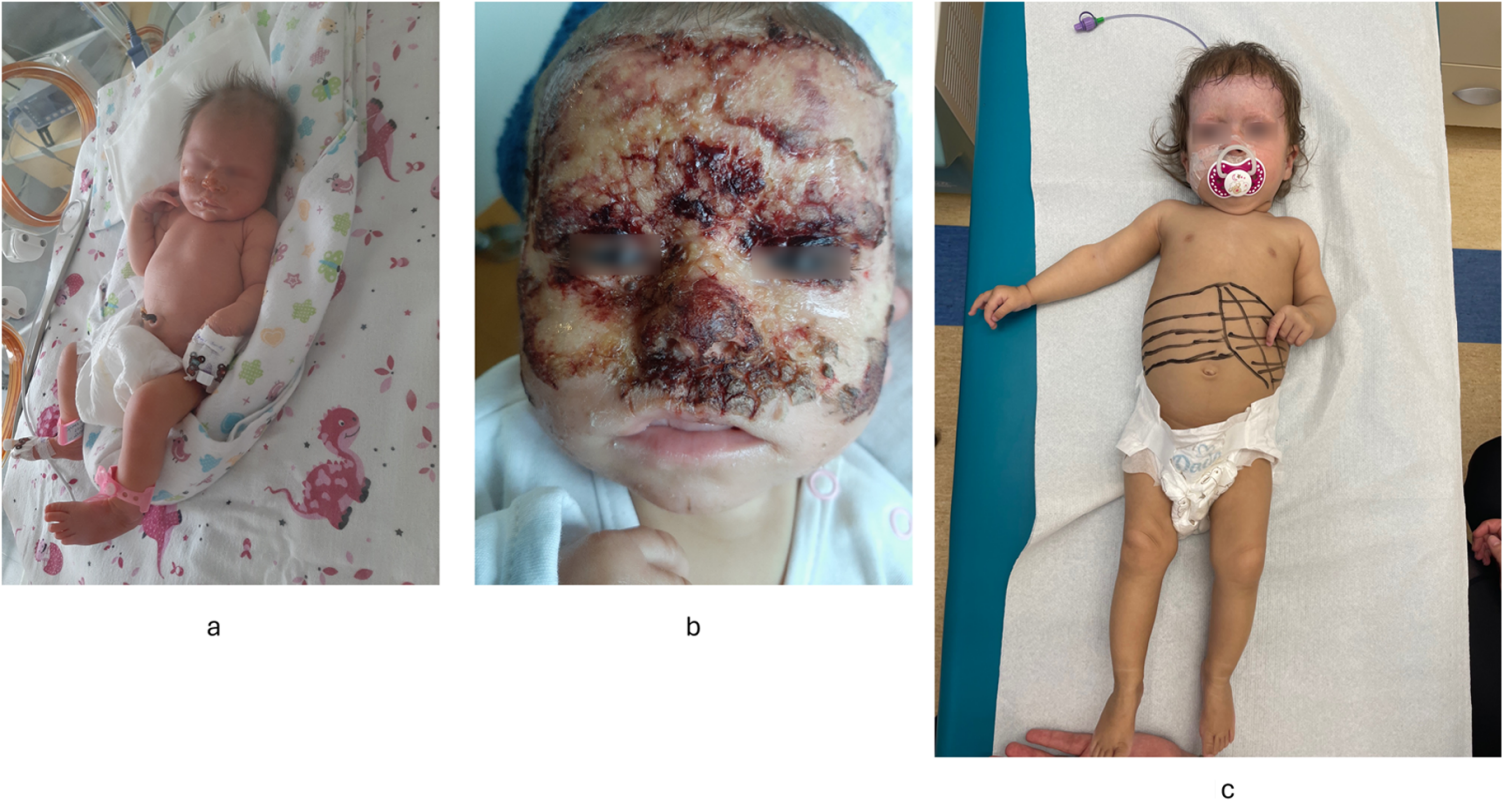
Results of physical examination. **(a)** Skin changes noted in the neonatal period. **(b)** Skin changes at 8 month of patient's age suggesting Staphylococcal Scalded Skin Syndrome. **(c)** Hepatosplenomegaly at 8 months of age.

The patient was the 1st child of nonconsanguineous Polish parents born at 36 weeks of gestation due to pregnancy complicated by an intrauterine growth restriction and oligohydramnios (noted in the 3rd trimester) with a birth mass of 2.240 g. From the first days of life, erythematous rash with skin erosions covered by scubs, on the face and hands, were observed (Figure 1a). Normal liver and spleen volume along with small kidneys with lack of corticomedullary differentiation on ultrasound (suggesting renal hipodysplasia) were noted. Laboratory results revealed a mild cholestasis with elevated serum GGT (no coagulopathy), and increased serum creatinine (Table 1). Echocardiography showed a large (7–9 mm) and hemodynamically significant atrial septal defect (ASD). Infectious causes of cholestasis (including HBV, HCV, CMV, EBV, HIV, PBV19, congenital syphilis, and *Toxoplasma gondii* infections) were excluded serologically; alpha-1-antitrypsin deficiency, cystic fibrosis, galactosemia were excluded as well. Ursodeoxycholic acid (UDCA) and fat-soluble vitamins were implemented leading to decrease of serum bilirubin concentration within one week (Table 1).

**Table 1:** Results of laboratory analyses.

	Reference values	4 day of life	12 day of life	8 months	16 months	24 months (2 y)	26 months	30 months (2.5 y)
Haemoglobin [g/dl]	0–2 weeks, 14.9–23.7; 2 weeks–2 months, 13.4–9.8; 2–6 months, 9.4–13; 6 months–1 year, 11.1–14.1; 1–2 years, 11.3–14.1; 2–6 years, 11.5–13.5	12.5	15.2	8.6	12.2	9.7	7.8	6.7
Platelets [tys/ul]	150–450	274	512	104	176	186	75	150
ALT [IU/L]	<18 months, *N* < 55/60; 18 months-12 years, boys, *N* < 40; >12 years, boys, *N* < 26	40	12	89	156	61	141	48
AST [IU/L]	<52	30	35	300	256	228	293	172
γ-GT [IU/L]	<200	530	243	220	247	164	234	334
BA [ng/ml]	<10	n.a.	n.a.	59.7	60	253	219	133
INR	<1.2	1.01	0.98	1,02	1.2	1.4	1.69	1.43
Total serum bilirubin [mg/dl]	<1.0	7.4	1.4	5.3	1.33	7.4	6.85	5.68
Direct bilirubin [mg/dl]	<1.0	1.5	0.6	3.2	0.6	5.3	6.28	4.37
Serum creatinine [mg/dl]	0.38–0.54	1.6	0.8	0.7	0.56	0.48	0.49	0.77
Cystatin C [mg/L]	<2	n.a.	3.11	3.1	2.73	2.3	n.a.	3.37
NT-proBNP [pg/ml]	<320	n.a.	n.a.	3,135	2,583	250	9,812	28,099
Liver (MCL) [mm]	Not applicable	normal	normal	75	85	95	105	105
Spleen [mm]	Not applicable	normal	normal	70	110	115	110	100

At the age of 8 months, the patient was hospitalized due to jaundice, failure to thrive and skin changes suggesting Staphylococcal Scalded Skin Syndrome (Figure 1b). At that time, hepatosplenomegaly was noted – liver was palpable 4 cm below the costal margin (in ultrasound: 8 cm in MCL) while the spleen was palpable 2 cm below the costal margin (in ultrasound: 7 cm in length) (Figure 1c). Small kidneys (5 cm) with hyperechoic renal cortex were observed. Laboratory results revealed normocytic anemia (requiring red blood cells transfusion), progressive cholestasis with elevated serum transaminases, mildly increased serum creatinine and cystatin C concentrations (Table 1). Heart failure was diagnosed supported by the results of chest radiography (enlarged heart), echocardiography (hemodynamically significant ASD, enlarged right atrium and ventricle) and high NT-proBNP value. UDCA treatment was continued while spironolactone and lisinopril were implemented. Nasogastric tube for feeding was also administered. Primary immune deficiency was excluded based on normal serum concentrations of immunoglobulins (G, A, M), high level of anti-HBs antibodies (after anty-HBV vaccinations), normal activity of NADPH oxidase and normal peripheral blood lymphocytes subpopulations (determined by flow cytometry). Organic acids analysis in urine (by GC/MS), serum acylcarnitine profile (by tandem mass spectrometry analysis), serum transferrin isoforms analysis as well as serum aminoacids were found normal. Mild increase of serum chitotriosidase activity of 448 nmol/ml/h (reference range: <150) was obtained, suggesting for a storage disorder. Subsequently, normal results of acid sphingomyelinase, β-glucocerebrosidase and acid lipase in dried blood spot (DBS) lead do exclusion of Gaucher disease, acid sphingomyelinase deficiency and lysosomal acid lipase deficiency, respectively. Due to an unknown aetiology of presented features and limited diagnostic possibilities, whole exome sequencing (WES) was commenced.

At the age of 16 months, the patient underwent cardiac surgery for a heart defect with closure of the ASD with a pericardial patch. The progression of hepatosplenomegaly was observed while laboratory analyses revealed decreasing cholestatic parameters with stable kidney function (Table 1).

**Figure 2:**
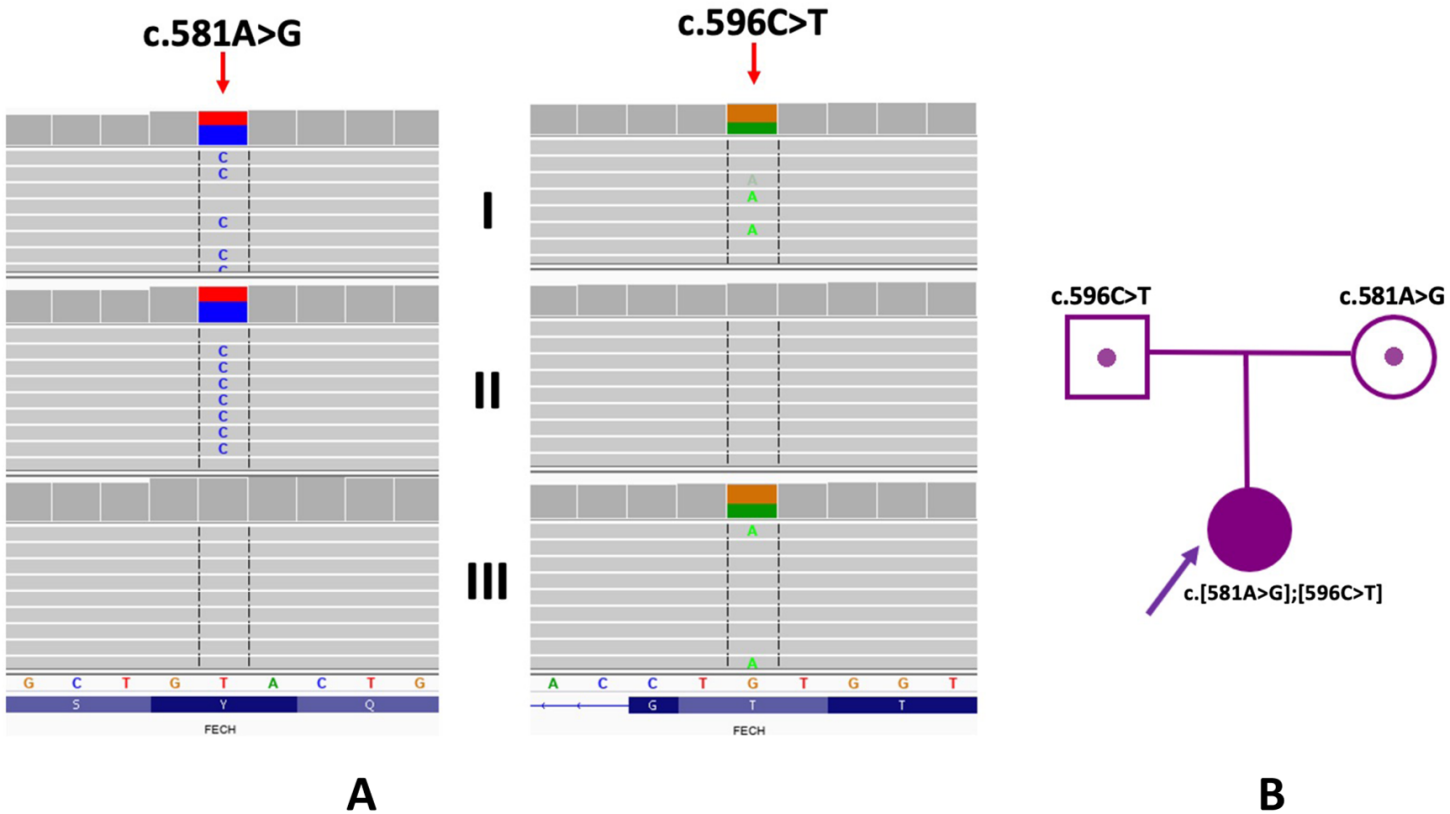
Genetic examination and family study of variants in the *FECH* gene. **(A)** WES results of proband and her parents (I - proband, II - mother; III - father). Integrative Genomic Viewer is presented. **(B)** Pedigree of the proband.

The proband was found to have the presence of two heterozygous missense variants in the *FECH* gene [NM_000140.5:c.581A>G, p.(Tyr194Cys) and NM_000140.5:c.596C>T, p.(Thr199Ile), Figure 2]. Segregation analysis of the *FECH* gene variants in the proband's family indicated an *in trans* arrangement. The proband exhibited a compound heterozygous configuration, while the parents showed carrier status. Specifically, the variant c.581A>G was identified in a heterozygous arrangement in the mother, and the variant c.596C>T in the proband's father. Neither of the identified *FECH* gene variants has been described in the literature. The presence of the c.581A>G variant was observed in three individuals (in a heterozygous arrangement) in the gnomAD control database (v4; https://gnomad.broadinstitute.org) and excluded (excluding proband's family samples) in the in-house database of >12,000 WES of Polish individuals. In-silico tools BayesDel_addAF, BayesDel_noAF, MetaRNN, REVEL predicted a pathogenic outcome for this variant. According to the American College of Medical Genetics and Genomics (ACMG), the variant was predicted as “likely pathogenic” (6 points; PM2, PP3_Strong).

The c.596C>T variant involves a change in a conserved nucleotide (PhyloP100 9.77). The c.596C>T variant was not found in the gnomAD control database (v4; https://gnomad.broadinstitute.org) and was present in one individual (in a heterozygous arrangement) in the in-house database of >12,000 WES of Polish individuals (excluding proband's family samples). In-silico tools BayesDel_addAF, BayesDel_noAF, MetaRNN, REVEL predicts a pathogenic outcome for this variant. According to the American College of Medical Genetics and Genomics (ACMG), the variant was predicted as “likely pathogenic” (6 points; PM2, PP3_Strong).

Since whole exome sequencing was used to identify variants, the region of the intronic polymorphism (hypomorphic allele) was not included in the study [PMID: 35054318].

Laboratory analyses revealed highly elevated total erythrocyte protoporphyrin (ePP) in red blood cells with predominant metal-free protoporphyrin IX (PPIX) confirming the genetic diagnosis of erythropoietic protoporphyria (Table 2).

**Table 2:** Results of porphyrins analysis.

	Results at 16 months of age	Results at 24 months of age	References
Quantification of free and zinc protoporphyrin in red blood cells			
Free protoporphyrin	52,135.7 nmol/L RBC	64,557.5 nmol/L RBC	<130
Zinc protoporphyrin	2,798 nmol/L RBC	n.a.	<1,200
Quantification of fecal porphyrin isomers			
Protoporphirin	2,062.6 nmol/g s.m.	1,075.7 nmol/g s.n.	0–100

At 2 years of age, the patient showed progression of hepatosplenomegaly with raising parameters of cholestasis (Table 1) as well as total erythrocyte protoporphyrin in red blood cells (Table 2).

At 26 months of age, the patient developed a respiratory syncytial virus-pneumonia complicated by an acute respiratory failure, requiring mechanical ventilation for about 14 days. During the disease course, a progressive coagulopathy, progressive heart failure (enlarged left ventricle with normal muscle thickness, enlarged left and right atrium, normal left ventricular systolic function) and deteriorating kidney function (Table 1) were observed. The patient required several red blood cells transfusions, intravenous vitamin K, and intensive diuretic treatment.

At 30 months of age the patient presented with two episodes of bleeding from esophageal varices. Deterioration of kidney and heart function were accompanied by oliguria, generalized edemas and pulmonary congestion. The patient finally died at 31 months of age due to multi-organ failure.

## 4 Discussion

This paper presents the case of a Polish infant with erytropoietic protoporphyria accompanied by other congenital anomalies, namely atrial septal defect and renal hypodysplasia. Both, progressive cholestatic liver disease (with subsequent congestive heart failure) and deteriorating renal function contributed to the patient's fatal outcome.

Clinical findings of liver disease in the course of EPP were reported in approximately 5%–20% of patients and were associated with loss-of-function *FECH* variants (5–7, 12, 13). Most common loss-of- function mutation is located in one allele of the FECH gene, which is mostly accompanied by a low expression *FECH* genetic variant (c.315-48C) in the other allele. [PMID: 35054318]. Since whole exome sequencing was used to identify variants, the region of the intronic polymorphism was not included in the study. Determination of the hypomorphic allele of the *FECH* gene seems to be necessary in patients with loss of function in one allele. In our patient, we observe 2 missense variants in a compound heterozygote pattern.

Interestingly, in the case presented here, two missense *FECH* mutations were found in a compound heterozygosity while the patient presented with progressive hepatocellular and cholestatic liver disease. This observation indicates the importance of other non-genetic factors in the pathogenesis of protoporphyric liver disease. Balwani et al. found elevated erythrocyte protoporphyrine IX (PPIX) levels positively correlating with an earlier age at onset and increased risk of liver dysfunction among patients with EPP (14). This fact truly corresponds with our patient's case as highly elevated PPIX levels with neonatal onset of liver disease were observed. Liver transplantation (LTx) constitutes the treatment for end-stage liver disease secondary to EPP but does not correct the underlying genetic defect (15, 16). Bone marrow transplantation can be curative and sequential liver and bone marrow transplantation has been successful in curing protoporphyric liver disease (17, 18).

Our case report highlights some similar pathogenic aspects between EPP and lysosomal storage diseases, namely the accumulation of non-degraded macromolecules. FECH deficiency leads to the accumulation of free protoporphyrin in erythrocytes, reticulocytes, erythroblasts, liver (hepatocytes, Kupffer cells and bile), plasma and skin (1, 19). Progressive hepatosplenomegaly was one the main clinical features observed in our patient and not reported in previous studies. Mildly elevated serum chitotriosidase activity, commonly used in the diagnostics of lysosomal storage diseases, was also observed in our patient, reflecting the accumulation within the reticuloendothelial system.

This is also an interesting report of the co-existence of EPP and renal hypodysplasia. While kidney disease is typical for acute intermittent porphyria (AIP), where porphyria-associated kidney disease occurs in more than 50% of the patients with symptomatic AIP, our case emphasizes that EPP and renal disease represents two separate disease entities (20).

## 5 Conclusions

Erytropoietic protoporphyria should be considered in patients with hepatosplenomegaly and cholestasis accompanied by skin damage.

The natural history of liver disease in the course of EPP could be determined by other factors, like the co-existence of congenital anomalies.

## Data Availability

The original contributions presented in the study are included in the article/supplementary material, further inquiries can be directed to the corresponding author/s.
